# The environmental stress sensitivities of pathogenic *Candida* species, including *Candida auris*, and implications for their spread in the hospital setting

**DOI:** 10.1093/mmy/myz127

**Published:** 2020-01-07

**Authors:** Helen Heaney, Juliette Laing, Linda Paterson, Alan W Walker, Neil A R Gow, Elizabeth M Johnson, Donna M MacCallum, Alistair J P Brown

**Affiliations:** Aberdeen Fungal Group, Institute of Medical Sciences, University of Aberdeen, Aberdeen, UK; NHS Grampian Central Decontamination Unit, Foresterhill Health Campus, Aberdeen, UK; NHS Grampian Central Decontamination Unit, Foresterhill Health Campus, Aberdeen, UK; Rowett Institute, University of Aberdeen, Aberdeen, UK; Aberdeen Fungal Group, Institute of Medical Sciences, University of Aberdeen, Aberdeen, UK; MRC Centre for Medical Mycology, University of Exeter, School of Biosciences, Exeter, UK; Mycology Reference Laboratory, PHE South West Laboratory, Southmead Hospital, Bristol, UK; Aberdeen Fungal Group, Institute of Medical Sciences, University of Aberdeen, Aberdeen, UK; Aberdeen Fungal Group, Institute of Medical Sciences, University of Aberdeen, Aberdeen, UK; MRC Centre for Medical Mycology, University of Exeter, School of Biosciences, Exeter, UK

**Keywords:** *Candida auris*, *Candida* pathogens, stress resistance, decontamination, hospital laundry

## Abstract

*Candida auris* is an emerging pathogenic yeast of significant clinical concern because of its frequent intrinsic resistance to fluconazole and often other antifungal drugs and the high mortality rates associated with systemic infections. Furthermore, *C. auris* has a propensity for persistence and transmission in health care environments. The reasons for this efficient transmission are not well understood, and therefore we tested whether enhanced resistance to environmental stresses might contribute to the ability of *C. auris* to spread in health care environments. We compared *C. auris* to other pathogenic *Candida* species with respect to their resistance to individual stresses and combinations of stresses. Stress resistance was examined using *in vitro* assays on laboratory media and also on hospital linen. In general, the 17 *C. auris* isolates examined displayed similar degrees of resistance to oxidative, nitrosative, cationic and cell wall stresses as clinical isolates of *C. albicans, C. glabrata, C. tropicalis, C. parapsilosis, C. krusei, C. guilliermondii, C. lusitaniae* and *C. kefyr*. All of the *C. auris* isolates examined were more sensitive to low pH (pH 2, but not pH 4) compared to *C. albicans*, but were more resistant to high pH (pH 13). *C. auris* was also sensitive to low pH, when tested on contaminated hospital linen. Most *C. auris* isolates were relatively thermotolerant, displaying significant growth at 47°C. Furthermore, *C. auris* was relatively resistant to certain combinations of combinatorial stress (e.g., pH 13 plus 47°C). Significantly, *C. auris* was sensitive to the stress combinations imposed by hospital laundering protocol (pH > 12 plus heat shock at >80°C), suggesting that current laundering procedures are sufficient to limit the transmission of this fungal pathogen via hospital linen.

## Introduction

A number of *Candida* species cause infections in humans. These yeasts are estimated to cause about 400,000 of the estimated two million life-threatening fungal infections that occur annually worldwide, generally in individuals undergoing surgery or with severely compromised immune systems.^1^ Indeed, the *Candida* genus is the fourth most common cause of hospital acquired bloodstream infections (candidemia) and the third most common cause in intensive care units.^2^ Historically, the majority of candidemia cases have been attributed to *Candida albicans*.^3^ However, the frequency of infections caused by non-*Candida albicans Candida* (NAC) species is increasing.^4,5^ The rising proportion of *Candida glabrata, Candida krusei* and *Candida guilliermondii* infections is thought to be due to their intrinsic resistance to certain antifungal drugs,^6,7^ such as fluconazole, which, when used routinely as a prophylaxis, can lead to selection of resistant species.^8^ Meanwhile, the prevalence of *Candida parapsilosis* outbreaks in intensive care units and premature neonates is probably due to this yeast's effective adherence to catheters and skin and the lipid rich environment favorable for its growth afforded by the frequent use of total parenteral nutrition (TPN) in this group.^9^

*Candida auris* has recently emerged as a global health concern.^10–12^*C. auris* was first isolated in South Korea in 1996, but was not identified as such at the time.^13^ Although present, *C. auris* infections were rare prior to 2009, when it was isolated from the ear canal of a patient in Japan and described as a new species.^14,15^ Evidence for *C. auris* as a cause of systemic infection in a health care setting was not documented until 2011 in South Korea.^13^ Since then, *C. auris* has spread around the globe at an alarming rate. March 2012 saw the first recorded outbreak in the Americas, in a hospital in Venezuela,^16^ this was quickly followed by the first outbreak in the USA in 2013.^16^ Sporadic cases also appeared in the UK from 2013,^17^ with the first UK outbreak in 2015.^18^ The propensity of C. *auris* to cause outbreaks in health care settings is now a major global health concern. The rapid emergence of *C. auris* has caused concern for several reasons. First, this species exhibits a propensity for patient-to-patient transmission in care settings, more so than other species of *Candida* and other pathogenic fungi.^19^ Amplified fragment length polymorphism (AFLP) analysis and genome sequencing has suggested that outbreak strains are highly clonal within geographical regions and distinct between continents.^18,20,21^ Based on these distinctions, *C. auris* isolates are considered to represent four phylogenetic clades,^14^ but recently a potential fifth clade has been described.^22^ Second, a significant proportion of *C. auris* strains display multidrug resistance. Approximately 90% of *C. auris* isolates are reported to be resistant to fluconazole, about 8% are resistant to amphotericin B, about 2% are resistant to echinocandins, and about 25% are resistant to more than one class of agent,^23^ with patterns of resistance closely linked to the phylogenetic clades.^24^ This drug resistance can lead to therapeutic failure. Third, in some settings *C. auris* is associated with an unusually high mortality rate compared to other *Candida* species. For example, whilst *C. albicans* is associated with ∼40% mortality in hospital patients,^25^ the mortality rate for *C. auris* in healthcare environments is 30–60%.^16,18,26^ Fourth, *C. auris* has proved difficult to identify using standard biochemical methods and consequently was often misidentified as other *Candida* species, particularly *Candida haemulonii*. This increased the chance of inappropriate treatment and, as a result, therapeutic failure.^26,27^

Currently, *C. auris* is the focus of a considerable research effort. This research has revealed that *C. auris* is closely related, phylogenetically, to *C. krusei, C. haemulonii*, and *C. lusitaniae*, all of which have been shown to exhibit resistance to azoles and/or amphotericin B.^14^ Genome sequencing combined with phenotypic analysis has revealed that *C. auris* shares many of the virulence factors and fitness attributes of *C. albicans*, such as multidrug efflux systems and cell wall remodeling pathways.^21,26,28^ Some *C. auris* strains exhibit salt tolerance, thermotolerance up to 42°C, as well as an aggregating phenotype that has been proposed to contribute to its efficient transmission.^28,29^

The persistence of *C. auris* in healthcare settings is also thought to have contributed to its emergence as a global health concern. *C. auris* has been found to particularly colonize the groin and axilla of infected and noninfected people, it was first isolated from aural cavities and has also been found colonizing the nostrils.^18,30^*C. auris* can also persist in patient rooms for extended periods of time, even after cleaning,^31^ on mattresses, chairs, windowsills, and areas around patient's beds.^18,30^ Quaternary ammonia products, such as Lysol and Virex II 256, have been shown to be relatively ineffective in killing C*. auris*,^32^ but evidence suggests that chlorine products are effective,^29,33–35^ and the Centers for Disease Control and Prevention (CDC) guidelines recommend use of disinfectants approved for use against *Clostridioides difficile*.^30^

We reasoned that resistance to certain environmental stresses might contribute to the spread of *C. auris* in healthcare environments. Hence, in this study we compared the stress phenome of *C. auris* to those of other pathogenic *Candida* species (*C. albicans, C. glabrata, C. tropicalis, C. parapsilosis, C. krusei, C. guilliermondii, C. lusitaniae*, and *C. kefyr*), revealing sensitivities to specific combinations of environmental stresses *in vitro.* The focus of most disinfection studies to date has been the removal of *C. auris* from hard surfaces or the prevention of transfer between individuals.^18,29,33–35^ Therefore, we extended our analyses to hospital bed linen, demonstrating that the environmental stresses imposed during hospital laundry procedures are effective in killing *C. auris*, as well as other pathogenic *Candida* species.

## Methods

### *Candida* clinical isolates

**Figure 1.**
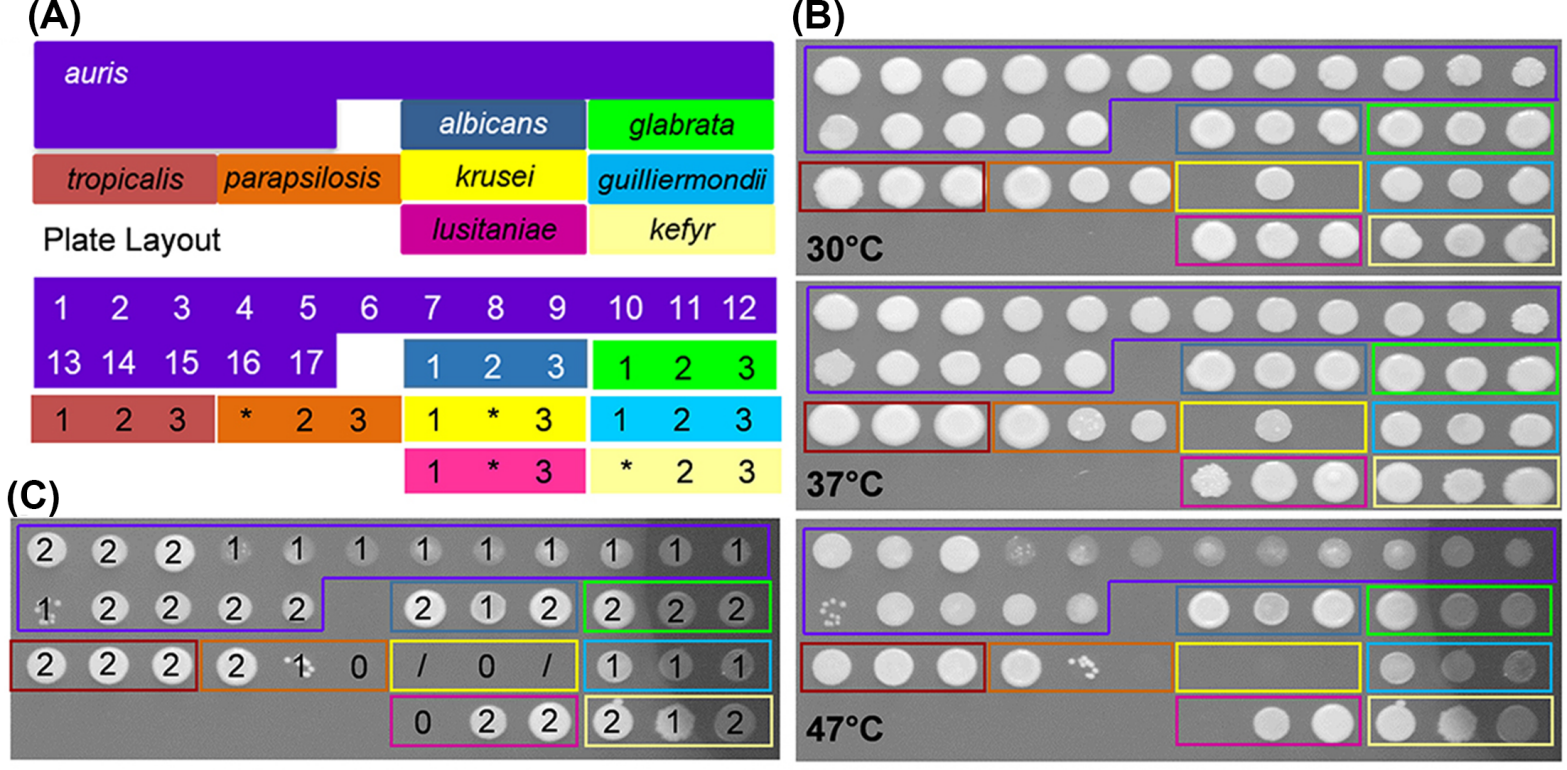
Comparison of *C. auris* thermotolerance with other *Candida* pathogens. **(A)** The microtiter plate layout of the *Candida* clinical isolates listed in Tables 1 and 2. Misidentified isolates are highlighted with an asterisk. **(B)** Growth of *Candida* isolates for 24 h on YPD agar at the stated temperatures. **(C)** Illustration of the semi-quantitative analysis of stress resistance, where 0 = no growth, 1 = less growth, 2 = ‘normal growth’ 3 = more growth, in comparison to the same isolate grown on YPD at 30°C, using the 47°C panel from (B) which illustrates one representative of six replicates. Data from the six replicate experiments were used to generate the averages presented in Tables S1 and S2. Note that while ‘*C. krusei*’ strains were included in the panel (yellow), for technical reasons these were excluded from our data analysis. This Figure is reproduced in color in the online version of *Medical Mycology*.

Seventeen *C. auris* isolates from the South Asian, East Asian, and South Africa clades were selected for analysis (Table 1). These isolates displayed different drug resistance profiles and aggregation phenotypes.^28^ For comparison with other *Candida* pathogens, we selected three isolates each of *C. albicans, C. glabrata, C. tropicalis, C. parapsilosis, C. krusei, C. guilliermondii, C. lusitaniae*, and *C. kefyr* from the collections of Donna MacCallum and Frank Odds (University of Aberdeen, UK) (Table 2). These 17 *C. auris* isolates and 24 other *Candida* isolates were arrayed in a microtiter plate format for stress resistance assays (Fig. 1).

**Table 1.** *Candida auris* strains.

					Mating		
Working name	Collection name	Isolated from	Clade	Aggregating	type	Drug resistance	Source
auris 1	Gow1715, 470026	blood	South Asian		a	FLZ, CAS	1
auris 2	Gow1716, 470027	blood	South Asian		a	FLZ, VORI, CAS	1
auris 3	Gow1717, 470028	blood	South Asian		a	FLZ, CAS	1
auris 4	Gow1718, 470029	blood	South Asian		a	FLZ, VORI, CAS	1
auris 5	Gow1719, 470030	blood	South Asian		a	FLZ, VORI, CAS	1
auris 6	Gow1720, 470031	blood	South Asian		a	FLZ, CAS	1
auris 7	Gow1721, 470032	blood	South Asian		a	FLZ, CAS	1
auris 8	Gow1722, 470033	blood	South Asian		a	FLZ	1
auris 9	Gow1723, 470034	blood	South Asian		a	FLZ, 5FC	1
auris 10	Gow1725, 470036	blood	South Asian		a	FLZ, CAS, 5FC	1
auris 11	Gow1726, 470037	blood	South Asian		a	FLZ, CAS, 5FC	1
auris 12	Gow1747, NCPF8980#9	blood	South African	Y	α	FLZ, CAS	2
auris 13	Gow1748, NCPF8984#15	unknown	East Asian	Y	α	FLZ	2
auris 14	Gow1750, NCPF8985#20	wound	South Asian		a	FLZ, ISAV, POSA, VORI, FLY, ANID, CAS	2
auris 15	Gow1749, NCPF13001#16	unknown	South Asian		a	FLZ	2
auris 16	Gow1751, NCPF13005#95	urine	South African	Y	α	FLZ, VORI, ANID, AMB, CAS	2
auris 17	VPCI479/P/13	blood	South Asian		a	FLZ	3

**Sources: (1)** Chakrabarti A. Incidence, characteristics and outcome of ICU-acquired candidemia in India. *Intensive Care Med*. 2015; 41: 285–295. doi: 10.1007/s00134-014-3603-2 **(2)** Dr. L. Johnson, Public Health England, Bristol **(3)** Sharma C, Kumar N, Meis JF, Pandey R, Chowdhary A. 2015. Draft genome sequence of a fluconazole-resistant *Candida auris* strain from a candidemia patient in India. *Genome Announc* 3(4): e00722-15. doi:10.1128/genomeA.00722-15.

**Table 2.** Other *Candida* strains.

Working name	Species	Collection name	Isolated from	Geographic source	Source	Biotyper results	Biotyper score	Chromogenic ID
Alb 1	*Candida albicans*	SC5314	blood		3	*Candida albicans*	2.17	*C. albicans*
Alb 2	*Candida albicans*	SCSB42457	blood	UK	2	*Candida albicans*	2.13	*C. albicans*
Alb 3	*Candida albicans*	AM2002/0089	catheter tip	UK	1	*Candida albicans*	2.189	*C. albicans*
Gla 1	*Candida glabrata*	SCS130399L	blood	UK	2	*Candida glabrata*	2.009	*C. glabrata*
Gla 2	*Candida glabrata*	SCS123636D	blood	UK	2	*Candida glabrata*	1.994	*C. glabrata*
Gla 3	*Candida glabrata*	AM2004/0091	central line	UK	1	*Candida glabrata*	1.955	*C. glabrata*
Tro 1	*Candida tropicalis*	SCS122443V	blood	UK	2	*Candida tropicalis*	2.102	*C. tropicalis*
Tro 2	*Candida tropicalis*	SCS76638K	blood	UK	2	*Candida tropicalis*	1.959	*C. tropicalis*
Tro 3	*Candida tropicalis*	AM2007/0111	IV catheter	UK	1	*Candida tropicalis*	2.049	*C. tropicalis*
Par 1	*Candida parapsilosis*	SCS5080820	blood	UK	2	*Candida glabrata*	1.8	*C. glabrata*
						*Candida tropicalis*	1.7	*C. tropicalis*
								*C. albicans*
Par 2	*Candida parapsilosis*	SCSXM70052	blood	UK	2	*Candida parapsilosis*	2.113	*C. parapsilosis*
Par 3	*Candida parapsilosis*	SCSBB425167	blood	UK	2	*Candida parapsilosis*	2.003	*C. parapsilosis*
Kru 1	*Candida krusei*	SCS71987M	blood	UK	2	*Candida krusei*	2.062	*C. krusei*
Kru 2	*Candida krusei*	SCS73972P	blood	UK	2	*Candida parapsilosis*	2.084	*C. parapsilosis*
Kru 3	*Candida krusei*	AM2019/001	femoral line tip	UK	1	*Candida krusei*	2.14	*C. krusei*
Gui 1	*Candida guilliermondii*	SCSBB418097	blood	UK	2	*Candida guilliermondii*	2.104	*C. guilliermondii*
								*C. albicans*
Gui 2	*Candida guilliermondii*	SCS74937K	blood	UK	2	*Candida guilliermondii*	2.127	*C. guilliermondii*
Gui 3	*Candida guilliermondii*	SCSMB042615	blood	UK	2	*Candida guilliermondii*	2.1	*C. guilliermondii*
Lus 1	*Candida lusitaniae*	SCS211362H	blood	UK	2	*Candida lusitaniae*	2.275	*C. lusitaniae*
Lus 2	*Candida lusitaniae*	SCS17999	blood	UK	2	not reliable identification	1.367	*C. lusitaniae*
								*C. krusei*
Lus 3	*Candida lusitaniae*	SCS401413	blood	UK	2	*Candida lusitaniae*	1.743	*C. lusitaniae*
Kef 1	*Candida kefyr*	J981143	unknown	USA	1	*Candida tropicalis*	2.262	*C. tropicalis*
Kef 2	*Candida kefyr*	81/017	unknown	unknown	1	*Candida kefyr*	1.765	*C. kefyr*
Kef 3	*Candida kefyr*	Y128	unknown	unknown	1	*Candida kefyr*	2.143	*C. kefyr*

**Sources: (1)** Dr. D. MacCallum, University of Aberdeen **(2)** Odds, F. C., Hanson, M. F., Davidson, A. D., Jacobsen, M. D., Wright, P., Whyte, J. A., et al. (2007). One year prospective survey of *Candida* bloodstream infections in Scotland. *J. Med. Microbiol.* 56, 1066–1075. doi: 10.1099/jmm.0.47239-0 **(3)** Gillum, A. M., Tsay, E. Y. H. & Kirsch, D. R. (1984). Isolation of the Candida albicans gene for orotidine-5′-phosphate decarboxylase by complementation of S. cerevisiae ura3 and E. coli pyrF mutations. *Mol Gen Genet* 198, 179–182.

The identity of the *C. auris* isolates had been confirmed by ITS sequence analysis.^36,37^ To confirm the identity of the other *Candida* isolates, these were analyzed by matrix-assisted laser desorption/ionization time-of-flight mass spectrometry (MALDI ToF MS)^38^ and by growth on chromogenic media (*Brilliance™ Candida Agar*, Oxoid, Hampshire, UK), which highlighted that some cultures were mixed. The initial species identification was confirmed for most isolates (Table 2). However, in a few cases, initial misidentifications were corrected by MALDI ToF MS: *C. krusei* 2 was in fact *C. parapsilosis; C. kefyr* 2 was *C. tropicalis*; and *C. parapsilosis* 1 was a mixture of *C. glabrata* and *C. tropicalis* (Table 2; Fig. S1). As strain identification was performed after the isolates were arrayed, misidentified *Candida* isolates were included in the stress panels, below (these are marked with an asterisk in the figures). *C. krusei* 1 and 3 were excluded from some assays (by washing these wells with 100% ethanol before replication onto test plates) because these isolates spread and overgrew neighboring isolates under certain growth conditions.

*Candida* strains were grown overnight in yeast-extract peptone dextrose (YPD) (1% yeast extract, 2% mycological peptone, 2% glucose)^39^ at the specified temperature for each experiment. Similar inocula of each isolate (based on optical density [OD]_600_), from early stationary phase liquid cultures, were aliquoted into a microtiter plate format (Fig. 1), glycerol was added to a final concentration of 20%, and replicate plates frozen at −80°C. Plates were then thawed at room temperature for 45 minutes before replica plating onto the specified media.

### Stress resistance on plates

To test the stress sensitivity of the *Candida* isolates, isolates were replica plated using an 8 × 12 prong replicator (Sigma-Aldrich, Dorset, UK) onto YPD agar supplemented with one of the following: 1 M NaCl; 0.6 M KCl; 25 mM succinic acid; 25 mM succinic acid plus 5 mM sodium nitrite (NaNO_2_); 1 mM *tert-*Butyl hydroperoxide (t-BOOH); 2.5, 5 or 7.5 mM hydrogen peroxide (H_2_O_2_); 100 µg/ml Calcofluor White (CFW); 100 or 150 µg/ml Congo Red (CR); or YPD adjusted to pH2, pH4, pH10, pH11, pH12, or pH13. Plates were incubated at 30°C, 37°C or 47°C for 24, 48, or 72 hours, as specified. Every experiment included a control YPD plate incubated at 30°C for 24 hours. Also, clinical isolates were exposed to the following stresses for 10 minutes in liquid suspension^40,41^ before plating on YPD: 0.5% chlorhexidine (Chlor); 0.5% sodium hypochlorite (NaClO); 2% alcohol ethoxylate (AlcEth). The growth of each isolate was rated qualitatively on a scale of 0 to 3, compared to the growth of the same isolate on YPD at 30°C in the absence of stress (where ‘normal’ growth was assigned 2, no growth was assigned 0, less growth was assigned 1, and more growth was assigned 3) (Fig. 1C). The average from six independent replicate experiments was then calculated to provide a semiquantitative measure of the effect of the stress upon growth.

### Stress resistance on hospital linen

To test the stress sensitivities of *Candida* clinical isolates on hospital linen, bedsheets were obtained from NHS Grampian, one of the regional health boards for the National Health Service Scotland, cut into required sizes and sterilized by autoclaving. The 41 *Candida* isolates were replica plated onto 12 × 8 cm rectangles of linen (in the pattern shown in Fig. 1) and, after inoculation, these sheets were laid onto YPD plates containing different stressors. After incubation for 48 hours at 30°C, the linen was removed to assess the growth of each isolate. Alternatively, where specified, individual *Candida* isolates were inoculated onto clean, sterilized 1 × 1 cm squares (approximately 1 × 10^6^ cells per square). To simulate patient soiling, 30 µl of test soil (Browne washer/disinfector test soil, Serris, France), a substance used to test washer efficiency by mimicking contamination by bodily fluids, was added to the squares after inoculation. Inoculated squares were then laid on YPD plates (containing different stressors, as above) and incubated at the relevant temperature for 1–3 days. The squares were removed, and cells were recovered by vigorously vortexing each square in 1 ml sterile water. The cells were harvested by centrifugation and resuspended in 1 ml water, then the OD_600_ of the cell suspension measured to estimate the amount of *Candida* recovered after growth on each inoculated linen square. While the degree of growth in the presence of stress varied, generally over 95% of those cells harvested after growth were viable, based on cfu assays.

## Results

### *C. auris* isolates generally display similar stress sensitivities to other *Candida* species

The *Candida* isolates were exposed to a range of individual stress conditions including growth at different temperatures (ranging from 30 to 47°C) and pH (2 to 13), cationic stresses (NaCl, KCl), oxidative stresses (H_2_O_2_, t-BOOH), nitrosative stresses (sodium nitrite), and cell wall stresses (Calcofluor White, Congo Red). The amount of growth on plates was estimated (Fig. 1), and the average from six experiments (two technical replicates in each of three biological repeats) was used to generate a semi-quantitative measure of the effect of a stress upon growth. These data are summarized in Table S1.

**Figure 2.**
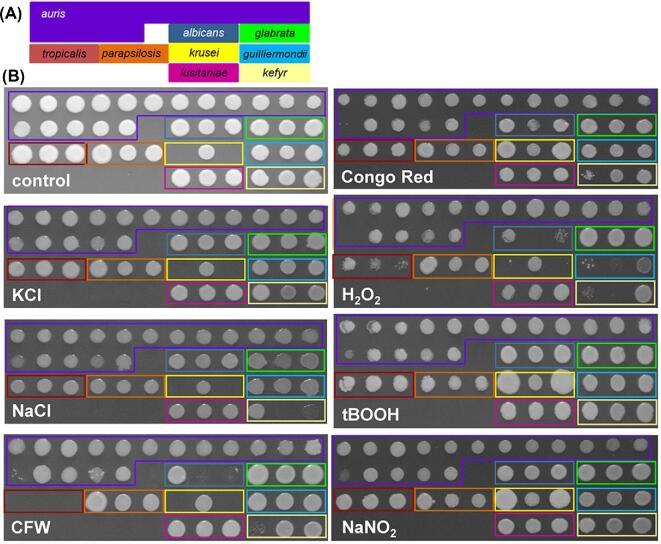
Resistance of *C. auris* and other *Candida* pathogens to individual environmental stresses. **(A)** The layout of the *Candida* clinical isolates to facilitate interpretation. **(B)** Growth of these *Candida* clinical isolates on YPD at 30°C for 24 h in the presence of the following stressors: no stress, control; 0.6 M KCl; 1 M NaCl; 100 µg/ml Calcofluor White, CFW; 150 µg/ml Congo Red; 7.5 mM H_2_O_2_; 1 mM t-BOOH); 25 mM succinic acid plus 5 mM NaNO_2._ Illustrates one representative of six replicates. This Figure is reproduced in color in the online version of *Medical Mycology*.

All of the *Candida* isolates tested grew well at 30 and 37°C, but the growth of a subset of *C. auris* isolates (4–13) was attenuated at 42°C and 47°C (Fig. 1B, Table S1). The *C. auris* isolates were generally resistant to the cationic, oxidative, nitrosative and cell wall stresses we examined, with the exception of *C. auris* 13 and 16 (Fig. 2; Table S1). *C. auris* 13 and 16 are from the East Asian and South African clades, respectively. Therefore, their sensitivities appear to be isolate-, rather than clade-, related. The stress resistance of most *C. auris* isolates we examined is consistent with recent data showing that some *C. auris* isolates are resistant to double the concentration of CFW and Congo Red used in this study and to similar levels of cationic and oxidative stress.^42^ Furthermore, the *C. auris* isolates generally displayed similar levels of resistance to those demonstrated by the other *Candida* isolates examined. However, all three *C. tropicalis* isolates and two *C. albicans* isolates (2 and 3) were particularly sensitive to Calcofluor White. Also, *C. glabrata* was particularly resistant to H_2_O_2_ (Fig. 2; Table S1).

**Figure 3.**
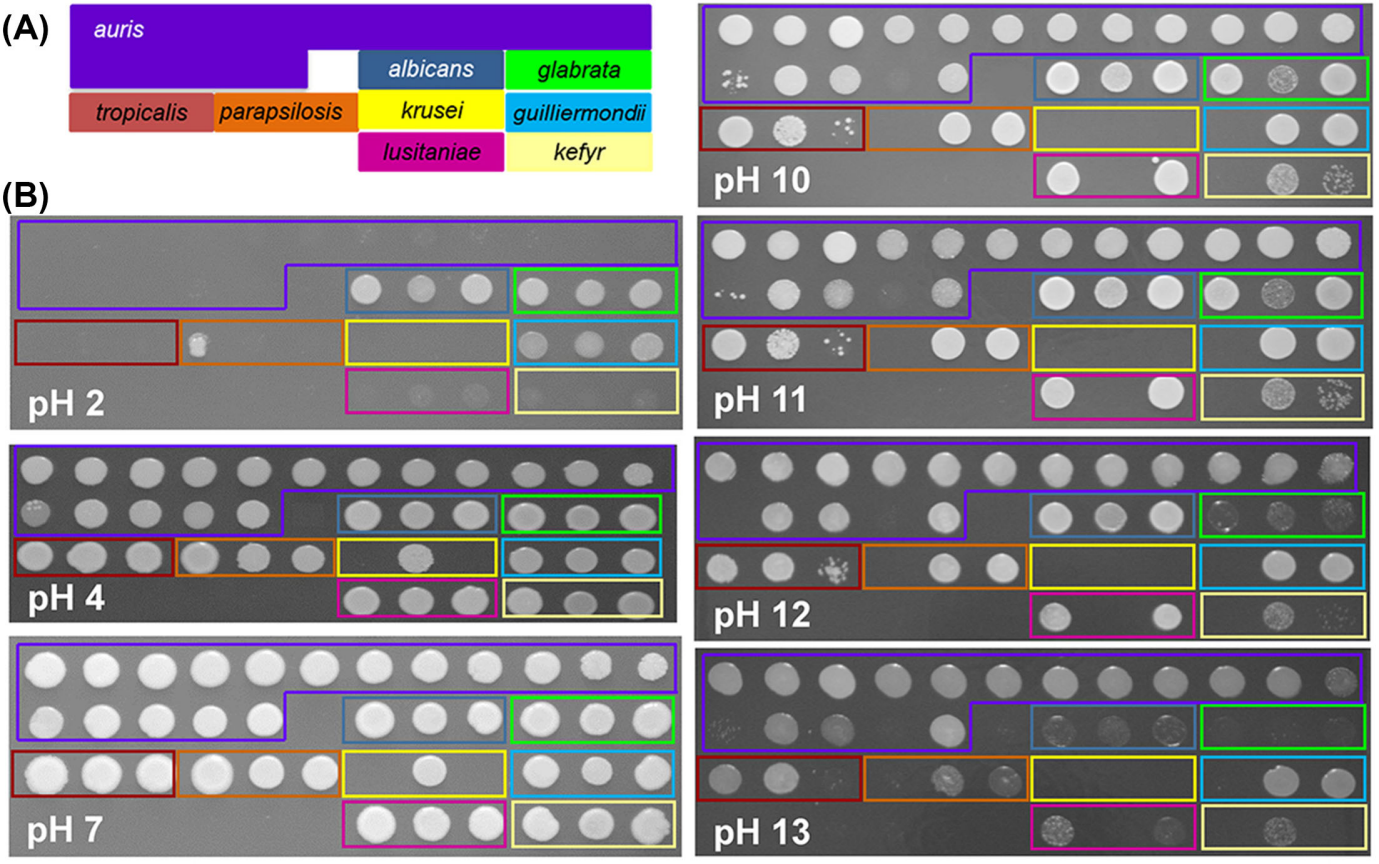
Resistance of the *C. auris* and other *Candida* isolates to pH stresses. **(A)** The layout of the *Candida* clinical isolates. **(B)** Growth of these *Candida* clinical isolates on YPD at 30°C for 24 h at the specified pH_._ Illustrates one representative of six replicates. This Figure is reproduced in color in the online version of *Medical Mycology*.

Significant differences in pH tolerance were observed between species (Fig. 3). *C. albicans* was tolerant to extremely low pH (pH 2) as reported previously,^43^ with *C .glabrata* and *C. guilliermondii* displaying similar degrees of acid tolerance. Interestingly, all *C. auris* isolates were unable to grow at acidic pH (pH 2) but, with the exception of isolates 13 and 16, were resistant to highly alkaline conditions (pH 13) (Fig. 3). Therefore, like other *Candida* pathogens, *C. auris* can tolerate a wide range of pH but, unlike most of the other species tested, it can grow at the high pH associated with detergents used during hospital laundering.^44^

### *C. auris* isolates and other *Candida* isolates are sensitive to certain combinatorial stresses

**Figure 4.**
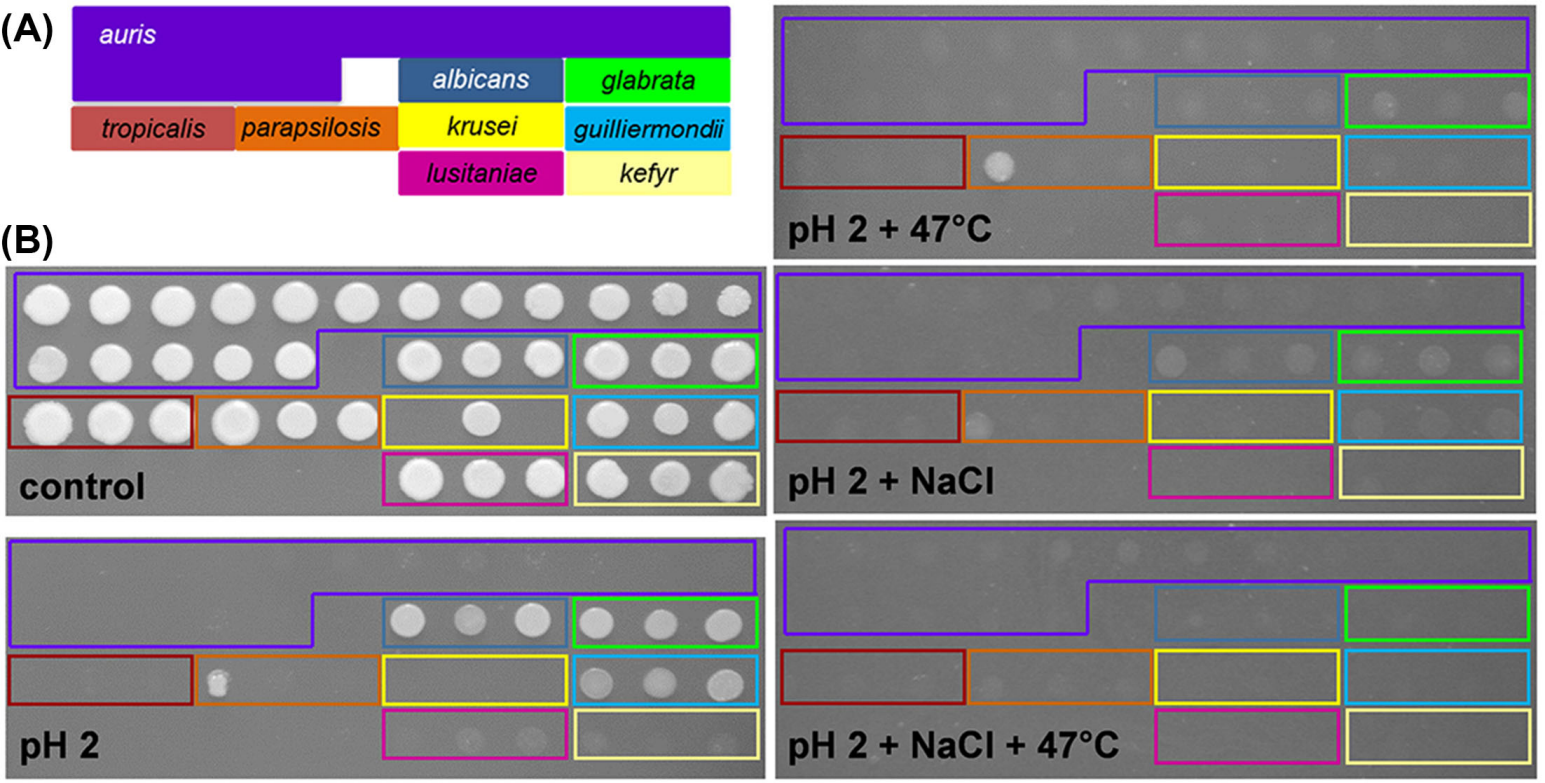
Resistance of the *Candida* isolates to combinations of salt, thermal and acid stress. **(A)** The layout of the *Candida* clinical isolates. **(B)** Growth of these *Candida* clinical isolates for 24 h at 30°C on unbuffered YPD (control) or, where specified, at pH2 with 1 M NaCl, or at 47°C. This Figure is reproduced in color in the online version of *Medical Mycology*.

**Figure 5.**
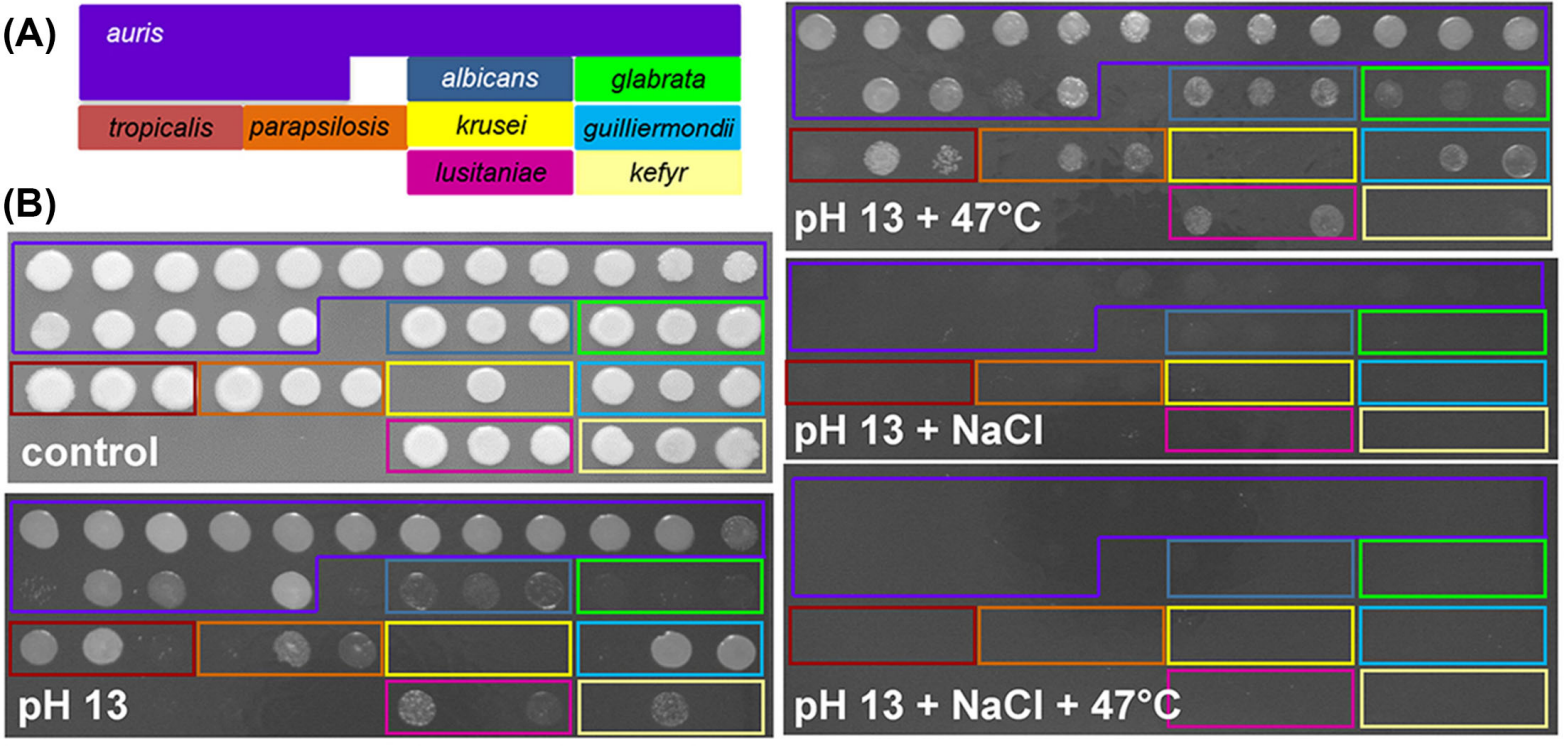
Resistance of the *Candida* isolates to combinations of salt, thermal and alkaline stress. **(A)** The layout of the *Candida* clinical isolates. **(B)** The growth of these *Candida* clinical isolates for 24 hours at 30°C on unbuffered YPD (control) or, where specified, at pH 13, with 1 M NaCl, or at 47°C. Illustrates one representative of six replicates. This Figure is reproduced in color in the online version of *Medical Mycology*.

*C. albicans* and *C. glabrata* are sensitive to certain combinations of stress, notably salt plus oxidative stress.^45^ Therefore, we compared the resistance of the *Candida* isolates to certain types of combinatorial stress, focusing initially on combinations of salt stress with those stresses to which some sensitivity had been observed, as described in the section above. We found that, unlike *C. albicans*,^46^*C. auris* isolates were relatively resistant to the combinatorial salt plus oxidative stress (Table S2). *C. parapsilosis* and *C. guilliermondii* were also resistant to this combinatorial stress, whereas *C. lusitaniae* and *C. kefyr* were sensitive (Table S2). Interestingly, all of the pathogenic *Candida* species tested were sensitive to combinations of NaCl with pH extremes, whether at pH 2 or pH 13 (Figs 4, 5), and where residual growth was observed at 30°C, all growth was inhibited when the temperature was raised to 47°C (Table S1). Therefore, combinatorial salt plus pH plus thermal stresses are particularly effective at killing *C. auris* and other pathogenic *Candida* species (Fig. 4).

### Stress resistance of *Candida* clinical isolates on hospital linen

**Figure 6.**
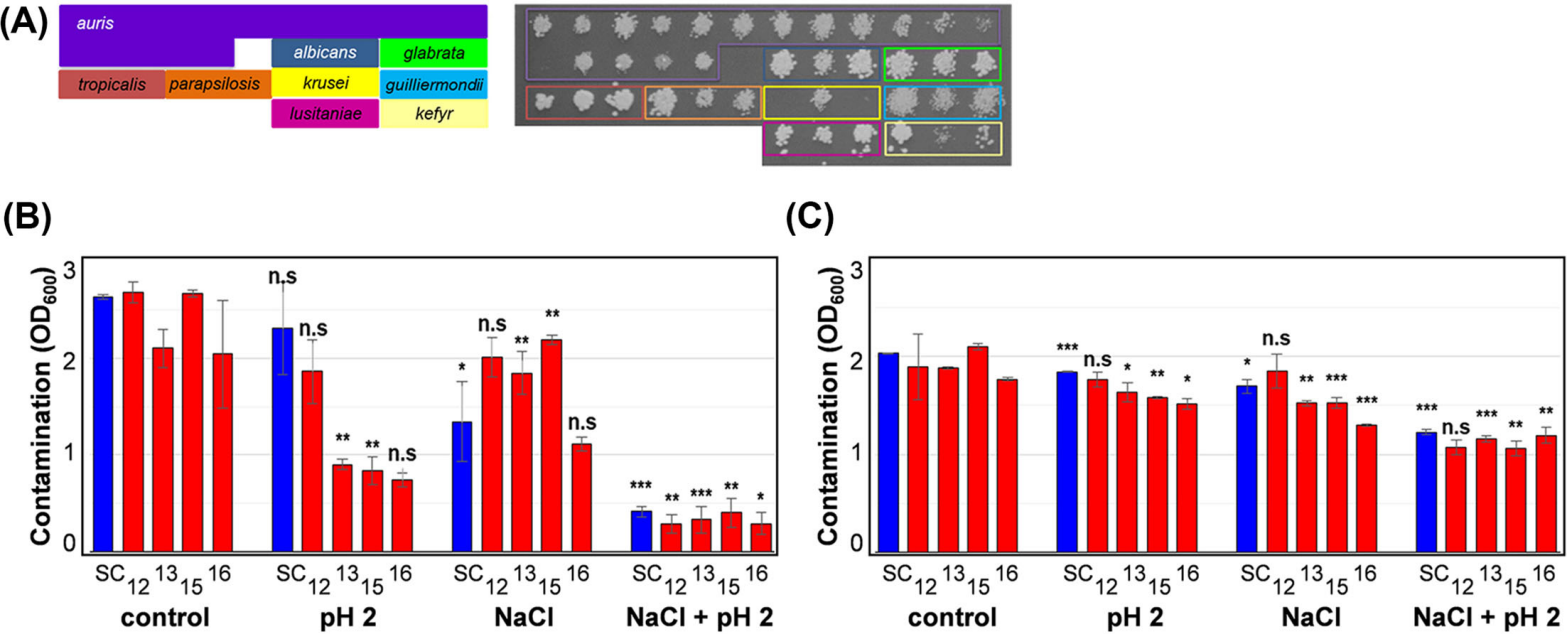
Resistance of the *C. auris* to combinations of salt and pH stress when contaminating ‘clean’ or ‘soiled’ hospital linen. **(A)** The layout of the *Candida* clinical isolates and their growth on YPD after contamination of clean linen. **(B)** Growth of selected clinical isolates on clean 1 × 1 cm linen squares in YPD in the presence or absence of stress (pH2 and/or 1 M NaCl) for 24 h at 30°C: *C. albicans* SC5314, SC; *C. auris* clinical isolates 12, 13, 15, 16. Growth was estimated by re-suspending the *Candida* cells in H_2_O and measuring the OD_600_/ml. **(C)** The resistance of these *Candida* isolates to combinations of acid and salt stress on soiled linen. Means and standard deviations from three independent replicate experiments are shown, and stressed isolates were compared to the relevant unstressed control using the student *t*-test: ns, not significant; *, *P* ≤ 0.05; **, *P* ≤ 0.01; ***, *P* ≤ 0.001; ****, *P* ≤ 0.0001. This Figure is reproduced in color in the online version of *Medical Mycology*.

It has been suggested that, in the hospital setting, *C. auris* might spread via contaminated fomites, such as bed linen.^18,30,47^ Therefore, we tested the stress sensitivities of the *Candida* isolates when they were contaminating hospital linen under controlled laboratory conditions. All of the isolates were inoculated onto linen squares, dried for 1 hour, laid on a YPD plate inoculated side up, and incubated at 30°C for 72 hours. The linen was then removed and the plate photographed (Fig. 6A), showing that most isolates remained viable and were able to grow under these conditions. However, despite testing a number of approaches, our attempts to quantify the degree to which each *Candida* isolate grew on the linen over YPD plates proved fruitless. The challenges were compounded by the aggregation phenotype of some *C. auris* isolates.^28^ Therefore, we estimated the growth of individual isolates on linen squares by resuspending the cells in water and measuring the resultant OD_600_.

Using this approach, we focused on five representative isolates: *C. albicans* SC5314 and *C. auris* isolates 12, 13, 15, and 16. First we examined them on ‘clean’ linen. The growth of three *C. auris* isolates (13, 15, 16) was attenuated at pH2 and none of these isolates were particularly affected by the salt stress, but the growth of all five isolates was inhibited by the combination of these two stresses (Fig. 6B). These data suggest that, for these *Candida* isolates, growth on linen does not confer resistance to combinatorial acid plus salt stress.

We then examined the impact of soiling upon the stress sensitivities of the *Candida* isolates on linen. Soiling enhanced the resistance of most clinical isolates to pH2 and/or salt stress, but particularly to combinatorial salt plus acid stress (Fig. 6C). Clearly this could be significant in the clinical setting.

### *Candida* resistance to clinically relevant disinfectants and laundry conditions

**Figure 7.**
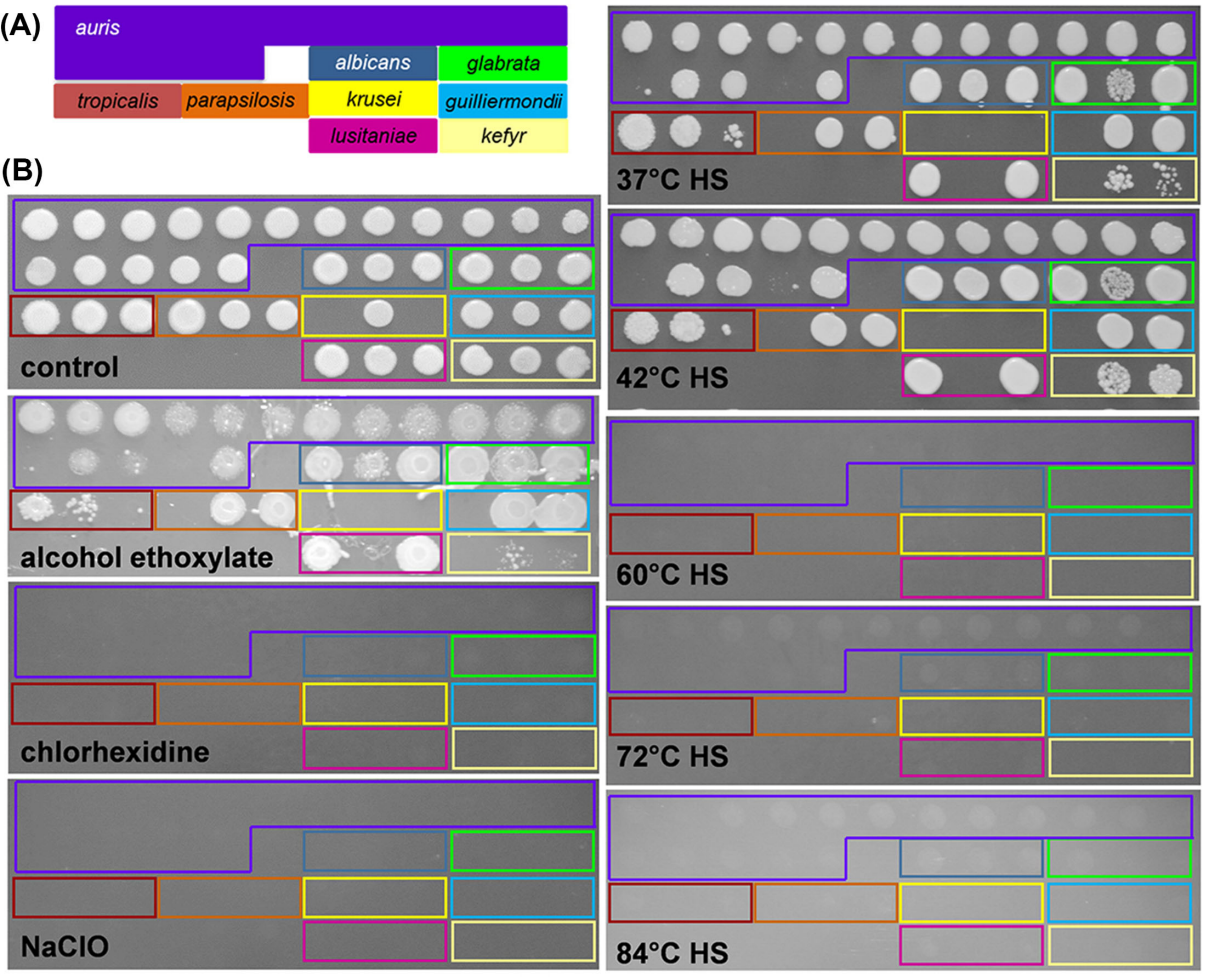
Resistance of the *Candida* isolates to hospital laundry-related environmental stresses. **(A)** The layout of the *Candida* clinical isolates. **(B)** The growth of these *Candida* clinical isolates for 24 h at 30°C on YPD after exposure to 0.5% chlorhexidine, 0.5% sodium hypochlorite (NaClO) or 2% alcohol ethoxylate for 10 min, or to the specified heat shock (HS) for 5 min. Illustrates one representative of six replicates. This Figure is reproduced in color in the online version of *Medical Mycology*.

Next we examined the impact of clinically relevant disinfectants and laundry conditions upon the full set of 41 *Candida* isolates. We tested the susceptibility of these isolates to chlorhexidine and sodium hypochlorite as these are frequently used to clean surfaces in patient rooms.^40,41,48,49^ The isolates were exposed to 0.5% chlorhexidine or sodium hypochlorite for 10 minutes before plating. These concentrations were based on standard cleaning recommendations and previous studies.^29,32,41,50,51^ All *Candida* isolates were sensitive to chlorhexidine and sodium hypochlorite under these conditions (Fig. 7B).

We also examined the effects of alcohol ethoxylate on the *Candida* isolates as this is the primary ingredient in detergents used in National Health Service (NHS) Scotland laundries (Dermasil Plus). The alcohol ethoxylate concentration we used (2%) was based on information provided by the Laundry Facility at the Foresterhill Healthcare Campus, NHS Grampian. Many of the clinical isolates, and most of the *C. auris* isolates in particular, were resistant to alcohol ethoxylate at 30°C (Fig. 7B). However, hospital laundry conditions also include rapid cycles of washing at high temperatures. For example, during the laundry process at the Foresterhill Healthcare Campus, the temperature is raised rapidly from 38°C to 52°C, and then to 81°C, 84°C, and 80°C. Each load spends at least 4.5 min above 80°C. Therefore, we tested the effects of a 5-min heat shock at different temperatures on the *Candida* isolates. Heat shock at 60°C or above killed all of the isolates examined (Fig. 7B).

**Figure 8.**
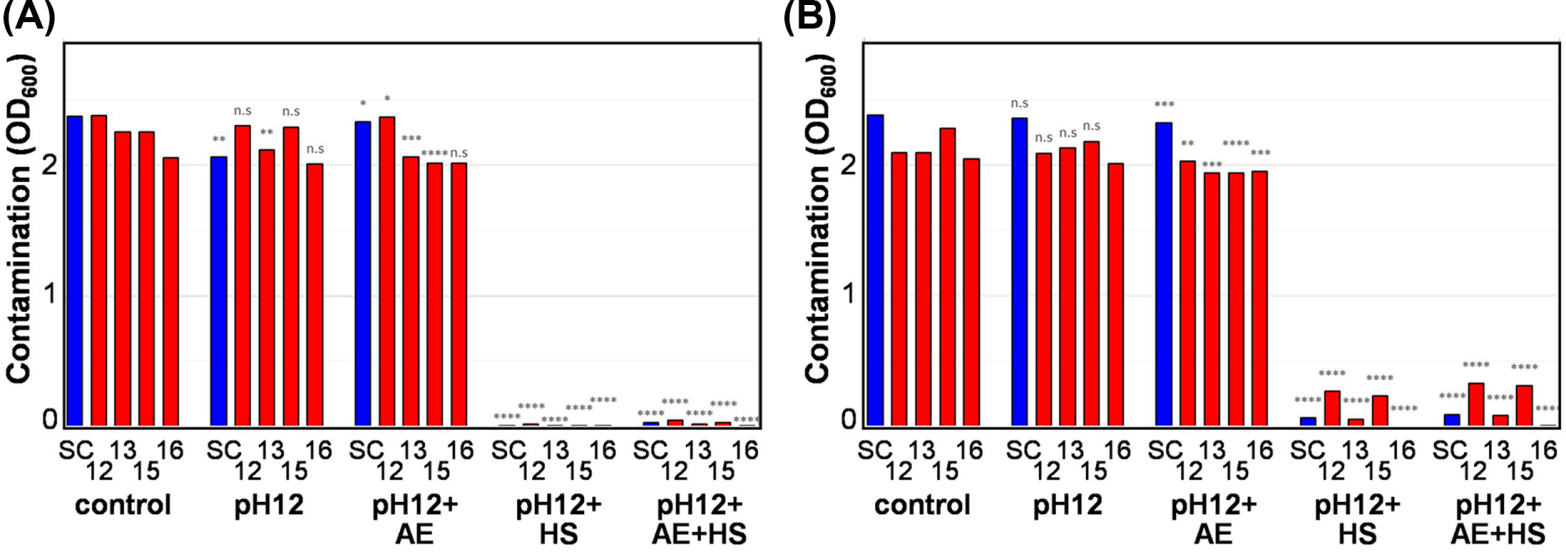
Resistance of the *C. auris* to a combination of laundry-related stresses on ‘clean’ or ‘soiled’ hospital linen. **(A)** The resistance of selected *Candida* isolates on clean linen to a combination of alkaline stress (pH12), alcohol ethoxylate (2%) and heat shock (84°C): *C. albicans* SC5314, SC; *C. auris* clinical isolates 12, 13, 15, 16. **(B)** Resistance of the same isolates on soiled linen to a combination of alkaline stress, alcohol ethoxylate and heat shock. Means and standard deviations from three independent replicate experiments are shown, and stressed isolates were compared to the relevant unstressed control using the student *t*-test: ns, not significant; *, *P* ≤ 0.05; **, *P* ≤ 0.01; ***, *P* ≤ 0.001; ****, *P* ≤ 0.0001. This Figure is reproduced in color in the online version of *Medical Mycology*.

We extended our analyses to hospital linen. We included alkaline pH because hospital laundry cycles can approach pH12. Consistent with our earlier finding (Fig. 5), *C. auris* isolates were resistant to pH12 when contaminating hospital linen (Fig. 8). However, the combination of alkaline stress with heat shock (84°C) was particularly effective at killing cells on ‘clean’ linen (Fig. 8A). However, some residual cells were observed on ‘soiled’ linen (Fig. 8B). We conclude that, while care might be required for badly soiled linen, hospital laundry conditions effectively kill the pathogenic *Candida* species we examined, including *C. auris*.

## Discussion

The pathogenic yeasts, *C. albicans* and *C. glabrata*, display relatively high levels of resistance to certain environmental stresses compared to less-pathogenic yeasts, such as *C. lusitaniae*, and this is thought to enhance their pathogenicity.^52,53^ We reasoned therefore that the ability of *C. auris* to persist in hospital environments and on individuals might be attributed, at least in part, to a relatively high resistance to environmental stresses compared with other *Candida* pathogens. With a view to obtaining a general, rather than an isolate-specific, view of their stress phenomes, 17 different *C. auris* isolates from different geographical locations were compared with at least two clinical isolates each of *C. albicans, C. glabrata, C. tropicalis, C. parapsilosis, C. guilliermondii, C. lusitaniae*, and *C. kefyr*.

Initially, we examined resistance to a range of individual environmental stresses. Few significant differences were observed between *C. auris* and the other *Candida* pathogens with respect to their thermotolerance or their resistance to salt, oxidative, nitrosative or cell wall stresses. This is consistent with a recent study that describes the importance of the stress activated protein kinase, Hog1, in mediating the resistance of *C. auris* to such stresses.^42^ Similar to other *Candida* isolates examined, *C. auris* isolates were tolerant over a broad pH range. However, *C. auris* was more resistant under alkaline conditions relative to *C. albicans* and *C. glabrata* (pH13). This is significant because hospital bleach is typically only pH12, and the activity range for quaternary ammonia products commonly used in health care environments is between pH3 and pH10.^54^ It has been reported that *C. auris* is less susceptible than *C. albicans* to 2% and 4% chlorhexidine^55^ and that *C. parapsilosis*, which has shown similar persistence levels on skin and environmental surfaces,^56^ is not killed by chlorine based cleaners after standard exposure times. In our hands, none of the *Candida* isolates grew after exposure to bleach or chlorhexidine *in vitro*. This confirmed and extended the findings of a recent report showing that *C. auris, C. albicans*, and *C. glabrata* are sensitive to sodium hypochlorite.^34,35,55,57,58^ However, it should be noted that, even after repeated application of chlorhexidine (a common skin disinfectant also used to disinfect surgical instruments), *C. auris* can still persist on patient skin.^18,34,59^ It has also been shown that *C. auris* can persist on hospital linen for several days.^60^ This suggests that sensitivity of *C. auris* to disinfection depends on the physical and physiological context.

We also examined combinatorial stresses because *C. albicans* is particularly sensitive to combinations of salt plus oxidative stress.^46^ We found that the *C. auris* isolates displayed similar sensitivity to combinations of salt, alkaline and thermal stress as the other *Candida* isolates we examined. Therefore, in general, our analyses of individual and combinatorial stresses did not support our initial working hypothesis that persistence of *C. auris* is attributable to high levels of environmental stress resistance compared to other *Candida* pathogens. The possible exception was the resistance of *C. auris* to combinatorial salt plus oxidative stress which could, in principle, compromise the ability of innate immune cells to kill this fungus.^46^ However, we reasoned that this would be unlikely to directly affect the transmission of *C. auris* in hospital settings.

Having examined *C. auris* and other *Candida* pathogens *in vitro*, we then tested the stress resistance of this pathogen when contaminating hospital linen. In general, the results were similar to those observed *in vitro*, where most *C. auris* isolates remained sensitive to combinatorial salt plus acid stress and the combination of alkali, alcohol ethoxylate and heat shock. However, the soiling of linen appeared to enhance resistance to these stresses. This observation, which is highly relevant to the clinical setting, strengthens the suggestion that the stress sensitivities of *C. auris* depends on the physiological context (above). For example, it is entirely conceivable that *C. auris* stress resistance might be enhanced during growth in mixed-species populations or biofilms.^29^ This would certainly be consistent with the fact that the growth conditions significantly affect the responses of *C. albicans* to environmental stresses.^61–63^

We also examined environmental stresses of direct relevance to hospital laundering, alcohol ethoxylate, thermal and alkaline stresses. Our data suggest that the heat shocks imposed during the laundry process promote the killing of the *Candida* pathogens we tested. However, it should be noted that our data do not exclude the possibility that nongrowing persister cells may survive these treatments.^59^ Furthermore, particular care might need to be taken with the washing process for heavily soiled laundry, as there appears to be an increased level of cells present on soiled linen after exposure to laundry conditions, compared to that of clean linen.

In conclusion, the profiles of *Candida* stress resistance observed in this study do not support our original working hypothesis that the nosocomial transmission of *C. auris* isolates is enhanced by their relatively high resistance to environmental stresses. Nevertheless, it would be prudent to test whether the stress resistance of *Candida* species, and *C. auris* in particular, is affected by other fabrics found in health care environments, such as healthcare professionals’ uniforms, some of which are washed by these professionals at home. This information, together with more accurate replication of the laundry process *in vitro*, would help to inform hospital laundering protocols in general.

## Supplementary Material

myz127_Supplemental_Files
