# Supplementary material for: The environmental stress sensitivities of pathogenic Candida species, including Candida auris, and implications for their spread in the hospital setting
Source: Med Mycol. 2020 Jan 7;58(6):744–55. doi: 10.1093/mmy/myz127 (PMC7398771; doi:10.1093/mmy/myz127)
Supplement: myz127_Supplemental_Files [file myz127_supplemental_files.zip › mm-2019-0220-File013.pdf]

Table S2: Combinatorial Stresses

The growth of each isolate in the presence of a stress was rated on a scale of 0 to 3, relative to the growth of the same isolate on YPD at 30°C in the absence of stress (where 'normal' growth was assigned 2, no growth was assigned 0, less growth was assigned 1, and more growth was assigned 3) (Figure 1B). The results from six replicate experiments are summarised. For abbreviations, see Materials & Methods.

Score	Meaning
3	More Growth
2	Same Growth
1	Less Growth
0	No Growth

	NaCl			KCl			pH2			pH4			NaCl + pH2		NaCl + H2O2		NaCl + pH12		pH13		NaCl + pH13	
Species	30°C	37°C	47°C	30°C	37°C	47°C	30°C	37°C	47°C	30°C	37°C	47°C	30°C	47°C	30°C	47°C	30°C	47°C	30°C	47°C	30°C	47°C
C. auris 1	2	2	1	2	2	2	0	0	0	2	2	1	0	0	2	2	1	0	2	2	0	0
C. auris 2	2	2	1	2	2	2	0	0	0	2	2	1	0	0	2	2	1	0	2	2	0	0
C. auris 3	2	2	1	2	2	2	0	0	0	2	2	2	0	0	2	2	1	0	2	2	0	0
C. auris 4	2	2	1	2	2	2	0	0	0	2	2	1	0	0	2	2	0	0	2	2	0	0
C. auris 5	2	2	1	2	2	2	0	0	0	2	2	1	0	0	2	2	0	0	2	2	0	0
C. auris 6	2	2	1	2	2	2	0	0	0	2	2	1	0	0	2	2	0	0	2	1	0	0
C. auris 7	2	2	1	2	2	2	0	0	0	2	2	1	0	0	2	2	1	0	2	2	0	0
C. auris 8	2	2	1	2	2	2	0	0	0	2	2	1	0	0	2	2	0	0	2	2	0	0
C. auris 9	2	2	1	2	2	2	0	0	0	2	2	1	0	0	2	2	0	0	2	2	0	0
C. auris 10	2	2	1	2	2	2	0	0	0	2	2	1	0	0	2	2	0	0	2	2	0	0
C. auris 11	2	2	1	2	2	2	0	0	0	2	2	1	0	0	2	2	0	0	2	2	0	0
C. auris 12	2	2	1	2	2	2	0	0	0	2	2	1	0	0	2	2	1	0	1	2	0	0
C. auris 13	1	1	1	1	1	1	0	0	0	2	2	0	0	0	0	0	0	0	1	0	0	0
C. auris 14	2	2	1	2	2	2	0	0	0	2	2	1	0	0	2	2	1	0	2	2	0	0
C. auris 15	2	2	1	2	2	2	0	0	0	2	2	1	0	0	2	2	1	0	1	2	0	0
C. auris 16	2	2	1	1	1	1	0	0	0	2	2	1	0	0	0	0	0	0	0	1	0	0
C. auris 17	2	2	1	2	2	2	0	0	0	2	2	1	0	0	2	2	1	0	2	2	0	0
C. albicans 1	2	2	1	2	2	2	2	2	0	2	2	2	1	0	1	1	0	0	1	1	0	0
C. albicans 2	2	2	0	2	1	1	1	1	0	2	2	1	1	0	0	0	0	0	1	1	0	0
C. albicans 3	2	2	1	2	2	2	2	2	0	2	2	2	1	0	1	1	0	0	1	1	0	0
C. glabrata 1	2	2	1	2	2	2	1	1	1	2	2	2	1	0	2	2	0	0	1	0	0	0
C. glabrata 2	1	1	1	2	2	2	1	1	0	2	1	1	1	0	1	1	0	0	1	1	0	0
C. glabrata 3	2	2	1	2	2	2	1	1	1	2	2	1	1	0	2	2	0	0	1	0	0	0
C. tropicalis 1	2	2	1	2	2	2	0	0	0	2	2	1	0	0	2	1	1	0	2	0	0	0
C. tropicalis 2	2	2	1	2	2	2	0	0	0	2	2	1	0	0	1	1	1	0	2	1	0	0
C. tropicalis 3	2	2	1	2	2	1	0	0	0	2	2	1	0	0	0	0	0	0	0	1	0	0
C. parapsilosis 2	2	2	0	2	1	1	0	1	0	2	1	1	0	0	2	1	1	0	1	2	0	0
C. parapsilosis 3	2	2	0	2	2	1	0	0	0	2	2	1	0	0	2	1	1	0	1	1	0	0
C. krusei 1	3	2	N/A	3	N/A	N/A	N/A	N/A	N/A	N/A	N/A	N/A	N/A	N/A	0	0	0	0	0	0	0	0
C. krusei 3	3	2	N/A	3	N/A	N/A	N/A	N/A	N/A	N/A	N/A	N/A	N/A	N/A	0	0	0	0	0	0	0	0
C. guilliermondii 2	2	2	0	2	2	1	1	1	0	2	1	1	0	0	2	1	1	0	2	2	0	0
C. guilliermondii 3	2	2	1	2	2	1	1	1	0	2	2	1	0	0	2	1	1	0	2	2	0	0
C. lusitaniae 1	2	2	0	2	2	1	0	0	0	2	2	1	0	0	0	0	0	0	1	1	0	0
C. lusitaniae 3	2	2	1	2	2	2	1	1	0	2	2	2	0	0	0	0	0	0	1	1	0	0
C. kefyr 2	1	1	0	1	1	1	0	0	0	2	2	1	0	0	0	0	0	0	1	0	0	0
C. kefyr 3	1	1	0	2	2	2	1	1	0	2	2	2	0	0	0	0	0	0	0	0	0	0
