# Supplementary material for: The environmental stress sensitivities of pathogenic Candida species, including Candida auris, and implications for their spread in the hospital setting
Source: Med Mycol. 2020 Jan 7;58(6):744–55. doi: 10.1093/mmy/myz127 (PMC7398771; doi:10.1093/mmy/myz127)
Supplement: myz127_Supplemental_Files [file myz127_supplemental_files.zip › mm-2019-0220-File012.pdf]

Table S1: Single Stresses

The growth of each isolate in the presence of a stress was rated on a scale of 0 to 3, relative to the growth of the same isolate on YPD at 30°C in the absence of stress (where ‘normal’ growth was assigned 2, no growth was assigned 0, less growth was assigned 1, and more growth was assigned 3) (Figure 1B). For abbreviations, see Materials & Methods.

Score	
3	More Growth
2	Same Growth
1	Less Growth
0	No Growth

H_2O_2*	
3	Growth at ≤ 7.5 mM
2	Growth at ≤ 5 mM
1	Growth at ≤ 2.5 mM
0	No Growth

Species	30°C	37°C	42°C	47°C	H_2O_2*	t-BOOH	NaCl	KCl	$NaNO_2$	Succinic	pH2	pH4	pH10	pH11	pH12	pH13	CFW	CR	AlkEth	Chlor	NaClO	HS 37 °C	HS 42 °C	HS 60 °C	HS 72 °C	HS 84 °C
C. auris 1	2	2	2	2	3	1	2	2	2	2	0	2	2	2	2	2	2	2	2	0	0	2	2	0	0	0
C. auris 2	2	2	2	2	3	1	2	2	2	2	0	2	2	2	2	2	2	2	2	0	0	2	2	0	0	0
C. auris 3	2	2	2	2	3	1	2	2	2	2	0	2	2	2	2	2	2	2	2	0	0	2	2	0	0	0
C. auris 4	2	2	1	1	3	2	2	2	2	1	0	2	2	2	2	2	2	2	2	0	0	2	2	0	0	0
C. auris 5	2	2	1	1	3	2	2	2	2	1	0	2	2	2	2	2	2	2	2	0	0	2	2	0	0	0
C. auris 6	2	2	1	1	3	2	2	2	2	1	0	2	2	2	2	2	2	2	2	0	0	2	2	0	0	0
C. auris 7	2	2	1	1	3	2	2	2	2	1	0	2	2	2	2	2	2	2	2	0	0	2	2	0	0	0
C. auris 8	2	2	1	1	3	2	2	2	2	1	0	2	2	2	2	2	2	2	2	0	0	2	2	0	0	0
C. auris 9	2	2	1	1	3	2	2	2	2	1	0	2	2	2	2	2	2	2	2	0	0	2	2	0	0	0
C. auris 10	2	2	1	1	3	2	2	2	2	1	0	2	2	2	2	2	2	2	2	0	0	2	2	0	0	0
C. auris 11	2	2	1	1	3	2	2	2	2	2	0	2	2	2	2	2	2	2	2	0	0	2	2	0	0	0
C. auris 12	2	2	1	1	3	2	2	2	2	1	0	2	2	2	1	1	2	1	2	0	0	2	2	0	0	0
C. auris 13	1	1	1	1	0	1	1	1	1	1	0	1	1	1	0	1	1	0	1	0	0	0	0	0	0	0
C. auris 14	2	2	2	2	3	1	2	2	2	2	0	2	2	2	2	2	2	2	1	0	0	2	2	0	0	0
C. auris 15	2	2	2	2	3	1	2	2	2	2	0	2	2	2	1	1	2	2	1	0	0	2	2	0	0	0
C. auris 16	2	2	2	2	3	1	1	1	2	2	0	2	0	0	0	0	1	1	1	0	0	0	0	0	0	0
C. auris 17	2	2	2	2	3	2	2	2	2	2	0	2	2	2	2	2	2	2	2	0	0	2	2	0	0	0
C. albicans 1	2	2	2	2	3	2	2	2	2	2	1	2	2	2	1	1	2	2	2	0	0	2	2	0	0	0
C. albicans 2	2	2	2	1	1	2	2	2	2	2	1	2	2	2	1	1	0	0	2	0	0	2	2	0	0	0
C. albicans 3	2	2	2	2	2	2	2	2	2	2	1	2	2	2	1	1	0	2	2	0	0	2	2	0	0	0
C. glabrata 1	2	2	2	2	3	2	2	2	2	2	1	2	2	2	0	1	2	2	2	0	0	2	2	0	0	0
C. glabrata 2	2	2	2	2	3	2	2	2	2	2	1	2	1	1	0	1	2	2	2	0	0	1	1	0	0	0
C. glabrata 3	2	2	2	2	3	2	2	2	2	2	1	2	2	2	0	1	2	2	2	0	0	2	2	0	0	0
C. tropicalis 1	2	2	2	2	2	1	2	2	2	2	0	2	2	2	2	2	0	0	1	0	0	2	2	0	0	0
C. tropicalis 2	2	2	2	2	1	2	2	2	2	2	0	2	2	2	2	2	0	0	1	0	0	2	2	0	0	0
C. tropicalis 3	2	2	2	2	1	2	2	2	2	2	0	2	1	1	1	0	0	0	0	0	0	1	1	0	0	0
C. parapsilosis 2	2	1	1	1	3	1	2	2	2	2	0	2	2	2	1	1	2	2	2	0	0	2	2	0	0	0
C. parapsilosis 3	2	1	1	0	3	1	2	2	2	2	0	2	2	2	1	1	2	2	2	0	0	2	2	0	0	0
C. krusei 1	3	N/A	N/A	N/A	NA	3	3	3	3	3	1	3	NA	NA	0	0	NA	1	NA	0	0	NA	NA	0	0	0
C. krusei 3	3	N/A	N/A	N/A	NA	3	3	3	3	3	1	3	NA	NA	0	0	NA	1	NA	0	0	NA	NA	0	0	0
C. guilliermondii 2	2	2	1	1	1	2	2	2	2	2	1	2	2	2	2	2	2	2	2	0	0	2	2	0	0	0
C. guilliermondii 3	2	2	1	1	2	2	2	2	2	2	1	2	2	2	2	2	2	2	2	0	0	2	2	0	0	0
C. lusitaniae 1	2	1	1	1	3	2	2	2	2	2	0	2	2	2	1	1	2	2	2	0	0	2	2	0	0	0
C. lusitaniae 3	2	2	2	2	3	2	2	2	2	2	0	2	2	2	1	1	2	2	2	0	0	2	2	0	0	0
C. kefyr 2	2	1	1	1	1	2	0	1	2	2	0	2	1	1	1	1	2	2	1	0	0	1	1	0	0	0
C. kefyr 3	2	2	2	2	3	2	1	2	2	2	0	2	1	0	0	0	2	2	1	0	0	1	1	0	0	0
